# Could Global Intensification of Nitrogen Fertilisation Increase Immunogenic Proteins and Favour the Spread of Coeliac Pathology?

**DOI:** 10.3390/foods9111602

**Published:** 2020-11-04

**Authors:** Josep Penuelas, Albert Gargallo-Garriga, Ivan A. Janssens, Philippe Ciais, Michael Obersteiner, Karel Klem, Otmar Urban, Yong-Guan Zhu, Jordi Sardans

**Affiliations:** 1 CSIC, Global Ecology Unit CREAF-CSIC-UAB, Bellaterra, 08193 Catalonia, Spain; a.gargallo@creaf.uab.cat (A.G.-G.); j.sardans@creaf.uab.cat (J.S.); 2 CREAF, Cerdanyola del Valles, 08193 Catalonia, Spain; 3 Global Change Research Institute, Czech Academy of Sciences, CZ-60300 Brno, Czech Republic; klem.k@czechglobe.cz (K.K.); otmar@brno.cas.cz (O.U.); 4 Research Group Plants and Ecosystems (PLECO), Department of Biology, University of Antwerp, B-2610 Wilrijk, Belgium; ivan.janssens@uantwerpen.be; 5 Laboratory of Climate and Environmental Sciences, Institute Pierre Simon Laplace (PSL), 91191 Gif-sur-Yvette, France; philippe.ciais@lsce.ipsl.fr; 6 Ecosystems Services and Management, International Institute for Applied Systems Analysis (IIASA), A-2361 Laxenburg, Austria; michael.obersteiner@gmail.com; 7 Key Laboratory of Urban Environment and Health, Chinese Academy of Sciences, Xiamen 361021, China; ygzhu@iue.ac.cn; 8 State Key Laboratory of Urban and Regional Ecology, Research Centre for Eco-Environmental Sciences, Chinese Academy of Sciences, Beijing 100085, China

**Keywords:** global intensification of N fertilisation, wheat, allergenic proteins, gluten proteins, coeliac pathology

## Abstract

Fertilisation of cereal crops with nitrogen (N) has increased in the last five decades. In particular, the fertilisation of wheat crops increased by nearly one order of magnitude from 1961 to 2010, from 9.84 to 93.8 kg N ha^−1^ y^−1^. We hypothesized that this intensification of N fertilisation would increase the content of allergenic proteins in wheat which could likely be associated with the increased pathology of coeliac disease in human populations. An increase in the per capita intake of gliadin proteins, the group of gluten proteins principally responsible for the development of coeliac disease, would be the responsible factor. We conducted a global meta-analysis of available reports that supported our hypothesis: wheat plants growing in soils receiving higher doses of N fertilizer have higher total gluten, total gliadin, α/β-gliadin, γ-gliadin and ω-gliadin contents and higher gliadin transcription in their grain. We thereafter calculated the per capita annual average intake of gliadins from wheat and derived foods and found that it increased from 1961 to 2010 from approximately 2.4 to 3.8 kg y^−1^ per capita (+1.4 ± 0.18 kg y^−1^ per capita, mean ± SE), i.e., increased by 58 ± 7.5%. Finally, we found that this increase was positively correlated with the increase in the rates of coeliac disease in all the available studies with temporal series of coeliac disease. The impacts and damage of over-fertilisation have been observed at an environmental scale (e.g., eutrophication and acid rain), but a potential direct effect of over-fertilisation is thus also possible on human health (coeliac disease).

## 1. Introduction

The demand for, and application of, nitrogen (N) fertilizer in cropland at a global scale has been continuously increasing. The global use of N fertilizers has increased substantially from 11.3 Tg N y^−1^ (0.9 g N m^−2^ y^−1^) in 1961 to 107.6 Tg N y^−1^ (7.4 g N m^−2^ y^−1^) in 2013 [1]. The data provided in the last International Nitrogen Initiative Conference indicated that the global consumption of N fertilizers increased 33% from 2000 to 2013 [2]. FAOstat data [3] indicated that the recent (2014–2018) intensification of N fertilisation at global and regional scales has affected most of the world, but with regional differences, with increases, from highest to lowest, of 29.1, 24.5, 17.6, 9.0, 5.4, 4.8, 4.1, 2.5 and 1.3% in eastern Asia, southern Asia, Latin America and the Caribbean, eastern Europe and central Asia, Sub-Saharan Africa, North America, western Asia, northern Africa and Oceania, respectively, and a decrease of 1.5% in western Europe [4]. Although continuous N fertilisation reduces nitrogen use efficiency on wheat yield production [5], this large increase in N fertilisation of wheat crops is the effective driver of the increased wheat yield [6,7]. This effect is especially important in wheat because it comes associated with a positive relationship between N fertilisation and wheat protein concentration [7,8].

**Figure 1:**
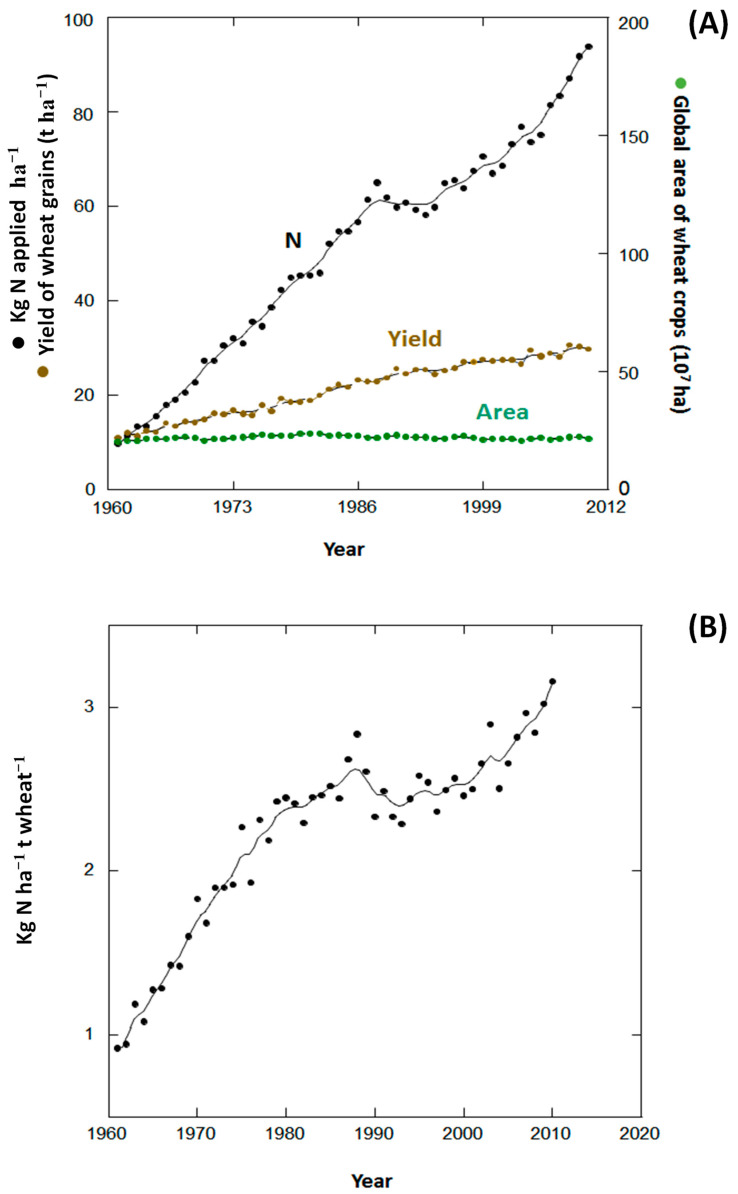
Global N-fertilisation rates (kg N ha^−1^ y^−1^) in wheat crops. Wheat grain yield (t ha^−1^ y^−1^) and global annual area of wheat crops (10^7^ ha) during 1961–2016 (1961–2010 for N-fertilisation rates) (**A**). Efficiency of N fertilisation (kg^−1^ N ha^−1^ per tonne of wheat grains (t wheat^-1^)) (**B**). Sources: [3,13,14,15]

Wheat (*Triticum* sp.) is currently the most widely planted crop and continues to be the most important food grain for humans [9]. Furthermore, despite a decrease of direct flour food products intake has occurred in some countries such as the United States of America, there is still a net increase in per capita annual wheat flour intake due to an additional flour intake from the extra flour used as food additive that has increased the net gluten annual intake per person from 4.1 kg in 1970 to 5.4 kg in 2000 [10]. Wheat crops currently cover an area of 217 × 10^6^ ha globally [11]. Global wheat yield in recent decades (1961–2015) has continuously increased despite representing a similar area of land (Figure 1) [3,12,13]. The annual amounts of N fertilizer applied to wheat crops have increased globally in the same period from approximately 10 to 100 kg N ha^−1^ y^−1^. This increase in N fertilisation is associated with an increase in the production of wheat grains and flour per hectare. The fertilisation (kg ha^−1^) to yield (t ha^−1^) ratio, however, increased from 0.9 to 3.1 kg N t grains^−1^ during 1961–2010, i.e., the yield-to-fertilisation ratio is now only 3.5-fold what it was 50 years ago.

The protein composition of wheat grains varies depending on genotype and environmental conditions, but wheat proteins are generally deficient in some fundamental amino acids, such as lysine and threonine [16]. Structural proteins of wheat grains are mostly albumins, globulins and amphiphilic proteins [16], whereas storage proteins are gliadins (monomeric proteins) and glutenins (polymeric proteins) [17]. N fertilisation generally influences the quantity and quality type of storage proteins (gliadins and glutenins) [18,19], but has little effect on structural proteins [20]. Martre et al. (2006) [21] modelled N-gliadin contents in wheat grains as a function of N partitioning among plant-protein groups and validated the model using 18 experimental studies. They observed a positive relationship between N-fertilisation rates and the amount of N allocated to gliadins after sowing.

Ingestion of wheat gluten can trigger several intolerances and allergic diseases, among which coeliac disease (CD) is the most widespread in humans [22]. The mean prevalence of CD in the general population in Europe and the United States of America (USA) is approximately 1% [23,24], with some regional differences, e.g., the prevalence of CD is as high as 2–3% in Finland and Sweden but is only 0.2% in Germany. The overall prevalence of CD is now clearly increasing everywhere. The prevalence of CD in the USA was only 0.2% in 1975 but increased 5-fold during the next 25 years [25]. The causes remain elusive but are likely linked to the environmental components of CD (e.g., changes in the quantity and quality of ingested gluten, patterns of infant feeding, the spectrum of intestinal infections and colonization by gut microbiota) [25].

Among the components of gluten, glutenins have been also associated with coeliac disease [26], but the group of gliadin proteins appears to be the primary cause of coeliac disease by gluten intake [22,27] in genetically susceptible individuals [28]. All three gliadin families, α/β, γ and ω, have been associated with allergic reactions to gluten and with the development of CD in humans [29,30,31,32,33,34,35,36,37]. The autoimmune response is due to the deamidation of glutamine residues in gliadins by human transglutaminase 2 (tTG2) produced in the gut mucosa [22,28,38,39]. These deamidated peptides can bind to histocompatibility leukocyte antigen (HLA) class II in some humans, which stimulates lymphocyte T cells and triggers an inflammatory response in the gut [22,23]. Most studies have reported that gliadins are most responsible for CD [29,30,31,32,33,34,35,36,37], but some studies have reported that the high-molecular-weight glutenins can also induce these autoimmune responses in some people [26,40,41]. Glutenins, however, are easily degraded by digestive enzymes, providing mostly di- and tripeptides, whereas gliadins are more resistant to enzymatic degradation, producing mostly oligopeptides that are the main cause of inflammatory responses [27]. We hypothesize that the increase in N fertilisation could be related to a potential increase of gluten in wheat grains and flours and thus to the spread of coeliac disease. More detailed information is now available from experimental data in field conditions including studies with a great variety of wheat genotypes growing in distinct areas of world. By gathering all this information, we aimed to analyse the gluten and gliadin concentrations in the grains and flour of wheat as a function of N-fertilisation levels and their potential association with higher coeliac disease prevalence at global scale.

## 2. Methods

We collected these data searching PubMed, ISI Web of Science and Google Scholar using the following terms spanning 1960–2019: coeliac, coeliac disease, nitrogen, fertilisation, gliadins, gluten, glutenin grain, wheat, and flour. We selected only studies providing information of the concentrations of these compounds in grain and/or flour and data on N-fertilizer doses that could be expressed in Kg ha^−1^ y^−1^. To obtain the data of the prevalence (percentage of coeliac cases in the total population) or incidence (new cases per 1000 inhabitants and year) of coeliac disease from studies with large and representative sets of population data, adjusted for age and sex, from 1961 to 2019, we have also searched PubMed, ISI Web of Science and Google Scholar using the following terms spanning 1960–2019: celiac, coeliac, celiac disease, coeliac disease, gluten, health, time, incidence and prevalence. We also used the FAO data: FAOSTAT http://www.fao.org/faostat/en/#data. (2019a) and the other sources cited in each figure caption. In the first bibliographic exploration, we gathered 172 articles. After data quality selection, we finally used the information provided by 47 articles (Table 1).

We examined the effects of the intensification of N fertilisation by a meta-analysis of the studies reporting the differences of total contents of gluten, total gliadins, α/β-gliadins, γ-gliadins, ω-gliadins and gliadin transcripts (operational taxonomic units) in wheat grains and/or flour under different levels of N fertilisation by calculating the response ratios from each study (Table 1), as described by Hedges et al. [42]. The natural-log response ratio (ln*RR*) was calculated as ln (*X_i_*/*X_n_*) *=* ln*X_i_* − ln*X_n_*, where *X_i_* and *X_n_* are the values of each observation in treated and control plants, respectively. The sampling variance for each ln*RR* was calculated as ln[(1/*n*_i_) × (*S*_i_/*X*_i_)^2^ + (1/*n*_n_) × (*S*_n_/*X*_n_)^2^] using the R package metafor 1.9–2 [43], where *n*_i_ and *n*_n_, *S*_i_ and *S*_n_ and *X_i_* and *X_n_* are the sample sizes, standard deviations and mean response values of the treatments and controls, respectively. The natural-log response ratios were determined by specifying studies as random factors using the *rma* model in metafor. The differences of contents of total gluten, gliadins, α/β-gliadins, γ-gliadins, ω-gliadins and gliadin transcripts in wheat grains and/or flour under different levels of N-fertilisation were considered significant if the 95% confidence interval of ln*RR* did not overlap zero. All statistical analyses were performed in R 3.6.0 (2019) (Copenhagen Business School, Copenhagen, Denmarck). We analyzed only the variables with >30 observations available at the global scale. We then examined the sensitivities of contents of total gluten, total gliadins, α/β-gliadins, γ-gliadins, ω-gliadins and gliadin transcripts in wheat grains and/or flour under different levels of N-fertilisation using REML estimation in the rma.unl model for metafor. We also analysed the relationship between the prevalence of coeliac disease and the per capita wheat intake at country level using a regression type II analysis conducted with the package lmodel2 [44] (R-Forge, Vienna, Austria).

**Table 1:** Responses of the concentrations of total gluten, total gliadins, α/β-gliadins, γ-gliadins and ω-gliadins and gliadin transcripts in wheat grains after an experimental increase in N fertilisation rates.

Bibliographic Source	Experimental Traits				Result
Reference	Site	Type of Experiment	Tested N-Fertilisation Rates (Kg N ha^−1^ y^−1^ if not Specified)	Genotypes	Changes in Concentrations in Grain and/or Flour in Response to Increasing N-Fertilisation
[45]	Experimental Farm Shiraz University (Iran)	Field	0, 120, 240, 360	Shiraz variety of winter wheat	Increases in gluten concentrations in grain
[46]	Bezek experimental station (Poland)	Field	0, 50, 80	*Triticum aesticum* ssp. *spelta*	Increases in total protein and gluten concentrations in grain
[47]	Two field areas of Tunis	Field	0, 67	Chili, Biskri, Mahmoudi, INRAT69, Karim, Razzak, Omrabiaa and Khiar varieties	Increases in gluten concentrations in grain
[48]	Experimental Station J. Hirschhorn (Argentina)	Field	0, 70, 140		Increases in gluten concentrations in grain
[49]	Upland crop experimental farm of National Institute of crop Science (Korea)	Field	25, 50, 75	Five Korean wheat varieties	Increases in gluten concentrations in flour. Increases in α + β-gliadin and decreases in ω and γ-gliadin concentrations
[50]	Replicates in five field sites in U.K.	Field	100, 200, 350	Five breadmaking wheat varieties (Cordial, Hereward, Malacca, Marksman and Xi19)	Increases in total gliadin concentrations in grain
[50]	Canada	Field	0, 100	Neepawa variety	Increases in total gliadin concentrations in grain
[51]	Canada	Field	0, 50, 100, 150, 200, 250, 300, 350, 400	Neepawa variety	Increases in total protein and total gliadin concentrations in grain
[30]	Eight different site sources	Field	0, 105, 165, 225	*Triticum aesticum* ssp. *spelta*	Increases of total epipodes expression of a-gliadin in grain
[52]	Plant Breeding Station of Sladkovicovo-Novy (Slovakia)	Field	120, 140	Winter wheat	Increases in total protein and gluten concentrations in grain
[53]	Experimental Station J. Hirschhorn (Argentina)	Field	0, 70, 140		Increases in total gluten concentrations in flour
[54]	Alava (Spain)	Field	0, 100, 140, 180	Soissons variety	Increases in total gliadin concentrations in grain
[55]	Spain	Pot experiment	37, 48 mg ammonium or nitrate per pot	Cezanne variety	Increases in total protein and gliadins concentrations in flour
[56]	Experimental field station Teramo University (Italy)	Field	50, 100, 150, 250	*Triticum turgidum* L. subsp*. durum*	Increases in total protein, gluten and gliadins concentrations in flour
[57]	Spain	Greenhouse	0, 22.2, 66.7, 200	Bobwhite variety	Increases in total, α, ω and γ-gliadin and total protein concentrations in flour
[58]	National Center of Irrigation Technology station (Spain)	Field	0, 120	Winter wheat	Increases in total gliadins concentrations in flour
[59]	Field (Sweden)	Field	0, 70, 140	Sport, Dacke, Dragon and Thasos varieties	Increases in total proteins and gliadins concentrations in flour
[60]	UK	Field	0, 40, 80, 120, 160, 200, 240	Option and Riband varieties	Increases in total proteins and gliadins concentrations in grain
[61]	Malice (Poland)	Field	0, 40, 80, 120	Tybalt variety	Increases in gluten concentrations in grain
[62]	Agricultural experimental Staion of University of Technology and Life Sciences of Minikowo (Poland)	Field	80, 120	Spring wheat	Increases in gluten concentrations in grain
[6]	Peterlauki research and Study Farm (Latvia)	Field	0, 60, 90, 120, 150, 180, 210, 240	Skagen variety	Increases in gluten concentrations in grain
[63]	Henan Agricultural University Experimental Satation (China)	Field	0, 90, 180, 270, 360, 450	Yumai and Lanko Aizao varieties	Increases in total gliadins concentrations in flour
[64]	Swadzim Experimental Station (Poland)	Field	0, 50, 100, 150	Durabon, Durabonus, Duraprimus and Rusticano varieties	Increases in gluten concentrations in flour
[65]	Lincoln Research Farm (New Zealand)	Field	0, 50, 100	Batten, Kotare, Oroua, Rongotea, Ruapuna and Tui varieties	Increases in total gliadins concentrations in flour
[66]	Mira (Italy)	Field	70, 120, 130, 160, 180, 200, 240	Biensur variety	Increases in gluten concentrations in flour
[67]	Two different sites (Austria)	Field	0, 180	Three varieties: Capo, Renan and Lindos	Increases in total, α, ω and γ gliadin concentrations in flour
[68]	Experimental Farm of Helsinki University (Finland)	Field	0, 110	Scandinavian, Kadett, Ruso and Reno wheat varieties	Increases in total proteins concentrations but not changes in gliadin concentrations in flour
[69]	Hungary	Field	30–300	Winter wheat	Increases in gluten concentrations in flour
[70]	Chile	Field	0, 220, 250		Increases in gluten concentrations in flour
[71]	France	Field	40, 60	Seedling from INRA	Increases in total gliadins concentrations in flour
[72]	Minokowo (Poland)	Field	0, 60, 90, 120	Zebra variety	Increases in gluten concentrations in flour
[73]	Field experimental Station of Mediterranean Agronomic Institute of Bari (Italy)	Field	30, 40, 50, 70	*Triticum turgidum* subsp. *durum*	Increases in total gluten concentrations in grain and flour
[74]	Brazil	Field	0, 50, 100, 150	Quartzo variety	Increases in gluten concentrations in grain
[75]	Experimental farm of INRA, Grignon, France	Field	40, 60, 120	Soissons variety	Increases in total gliadin concentrations in grain
[76]	Research field sation of Faculty of Agriculture (Croatia)	Field	0–194	Marija and Soissons varieties	Increases in gluten concentrations in grain
[77]	Rothamsted Research station (UK)	Field	100, 200, 350	Cordiale, Hereward, Istabraq, Malacca, Marksman and Xi 19 varieties	Increases in γ-gliadin gene expression
[78]	Rothamsted Research station (UK)	Field	100, 200, 350	Cordiale, Hereward, Istabraq, Malacca, Marksman and Xi 19 varieties	Increases in ω-gliadin gene expression
[17]	Germany	Field	0, 40, 120, 180, 200	Dozent, Monopol, Rektor, Apollo, Ares, Astron, Basalt, Bussard, Herzog, Ignaz, Kanzler, Monopol, Obelisk, Sperber varieties	Increases of α/β -gliadin, ω- and γ-gliadins, total gliadins and gluten concentrations in flour
[79]	Johann Heinrich von Thunen-Institute, Federal Research Institute for Rural Areas, Forestry and Fisheries, in Braunschweig, Germany	Field	84, 168	Batis variety	Increases of α/β -gliadin, ω- and γ-gliadins, total gliadins and gluten concentrations in flour
[80]	Research Station of Warmia and Mazury University (Poland)	Field	80, 120	*Spring triticale* cv. Andrus	Increases of α/β -gliadin, no clear effects on ω- and γ-gliadins in grain
[81]	Research Station of Warmia and Mazury University (Poland)	Field	80, 120	*Spring triticale* cv. Andrus	Increases of total gliadins concentrations in in grain
[82]	Uhrusk Experimental Station belonging to the University of Life Sciences in Lublin (Poland)	Field	90, 150	Opatka variety	Increases in gluten concentrations in grain
[83]	Fields research stations of Idaho and Monatana state Universities (USA)	Field	168, 224, 280	Spring wheat	Increases in gluten concentrations in flour
[84]	Futterkamp and Sonke-Nissen-Koog Northern Germany	Field	220, 260	Tobak and Asano varieties	Increases in total gliadin and gluten concentrations in flour
[85]	Grains Research Centre Kragujevac (Serbia)	Field	60, 90, 120		Increases in gluten concentrations in grain
[86]	China Agricultural University Research Center field station, Hebei province, China	Field	180, 240	Zhongmai variety	Increases in gluten concentrations in grain
[87]	Chongzhou and Renshou experimental stations of Sichuan Agricultural University, China	Field	0, 75, 150, 225	Shumai 969, Shumai 482, Chuannong 16 and Mianmai 51 varieties	Increases of total, α/β-gliadin and ω-gliadins and gluten concentrations, no clear effects on ω-gliadins in flour
[88]	Germany	Pot experiment	0.25, 1.0 and 2.5 g N/pot	Privileg variety	Increases of total gliadin concentrations

## 3. Increasing Gluten and Gliadin Contents with N-Fertilisation

**Figure 2:**
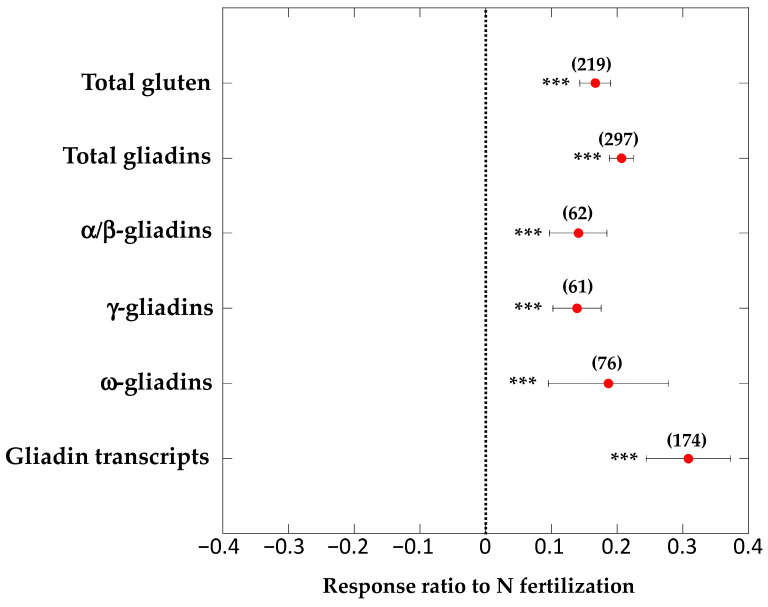
Response ratios (±95% CI) of contents of total gluten, total gliadins, α/β-gliadins, γ-gliadins and ω-gliadins and gliadin transcripts in wheat grains after an increase in N fertilisation. See Table 1 and References therein for the sources. The number into parenthesis indicates the number of studies. *** *p* < 0.0001.

**Figure 3:**
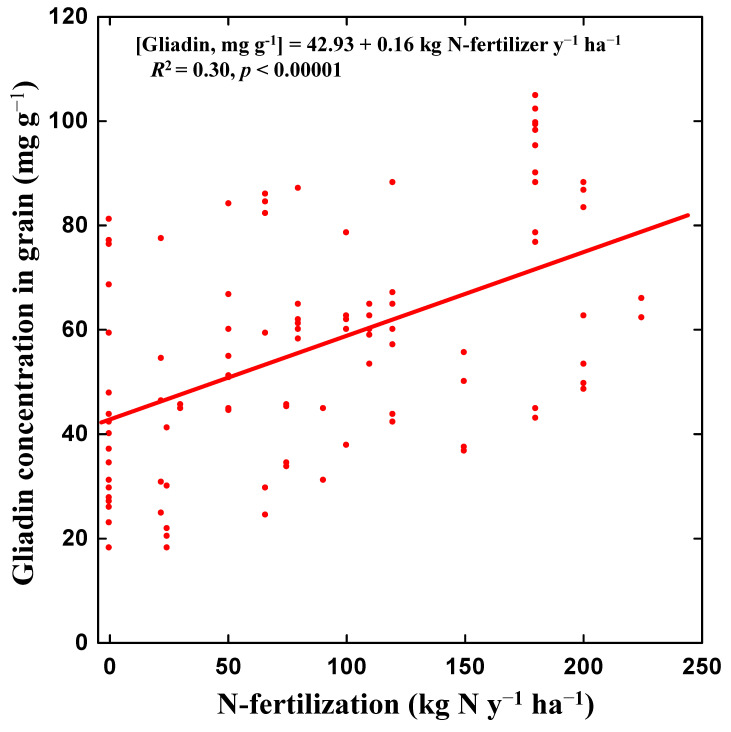
Increases in total gliadin contents in wheat grains as a function of the increases in N-fertilisation rates (kg N ha^−1^ y^−1^). See Table 1 for the sources.

Our meta-analysis found that the increase in N-fertilisation rates was associated with increased content of total gluten, total gliadins, α/β-gliadins, γ-gliadins, ω-gliadins and gliadin transcripts in wheat grains (Figure 2). Our analyses also identified a significant relationship (*R*^2^ = 0.30, *p* < 0.001) between the increase in N fertilisation and the increase in total gliadin content in wheat grains (Figure 3). Although the analysis has been conducted with very different genotypes of wheat growing under different soil types and climates, and therefore under very diverse conditions, the level of N fertilisation explained 30% of the change in gliadin content. These results are consistent with several studies observing a positive link of nutrient availability with the expression of gliadin genes [19,77,78,89] and all gluten proteins [17,90,91,92]. Furthermore, the results are also consistent with common farmer knowledge on protein concentration in wheat grain being strongly affected by N availability, which leads farmers to adjust the level of nitrogen fertilisation to obtain the required protein concentration in grain for bread making [93].

**Figure 4:**
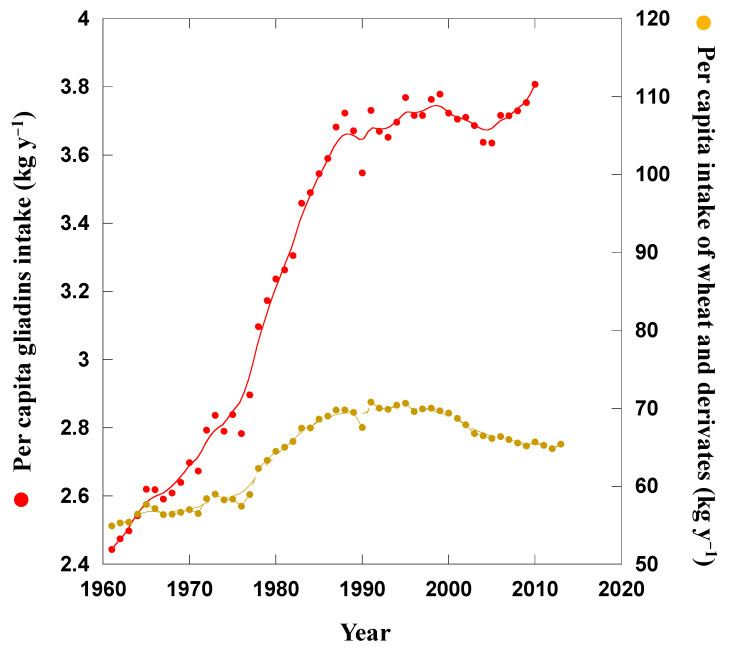
Per capita gliadin intake (g y^−1^) and per capita wheat and derivates intake (kg y^−1^).

The per capita annual increase in gliadin intake from wheat and derived foods during 1961–2010 was estimated to be approximately 1.4 kg y^−1^ (+58% ± 7.5%) (Mean ± SE; Figure 4). This estimation took into account the annual intake of wheat and derivatives at the global scale, [3] the increase in N fertilisation in wheat crops and the relationships between N fertilisation and gliadin increase (Figure 3 and Table 1). The increase in N-fertilisation from approximate 10 to 100 kg N ha^−1^ corresponded to an increase in gliadin contents in grains/flour from 44 to 59 mg g^−1^, respectively) (Figure 3 and Table 1).

## 4. Increased Prevalence of Coeliac Disease

Part of the increase in CD prevalence in populations in recent decades has frequently been attributed to improved diagnosis [94], with some studies suggesting that the increase in diagnoses was due to increased awareness [95]. Some studies of populations over time, however, have reported an actual increase in CD development in recent decades beyond the improvement of diagnostic efficiency [96]. The increase in diagnosed cases of new coeliac patients may thus be due to more efficient diagnosis and higher awareness, but also to changes in environmental variables associated with this increase in the percentage of a population affected by CD. Our study provides good evidence of a strong potential increase in the average human intake of gliadins by associating the changes in global per capita intakes of wheat and derivatives with the empirical effects of a global 10-fold increase in intensification of N fertilisation during 1961–2010. The contents of digested peptides derived from gliadin in the gut have been demonstrated to be a determinant for the appearance of autoimmunological responses and CD manifestation [97,98], and the amount of gluten/gliadin necessary to trigger CD in susceptible people can vary [99].

**Figure 5:**
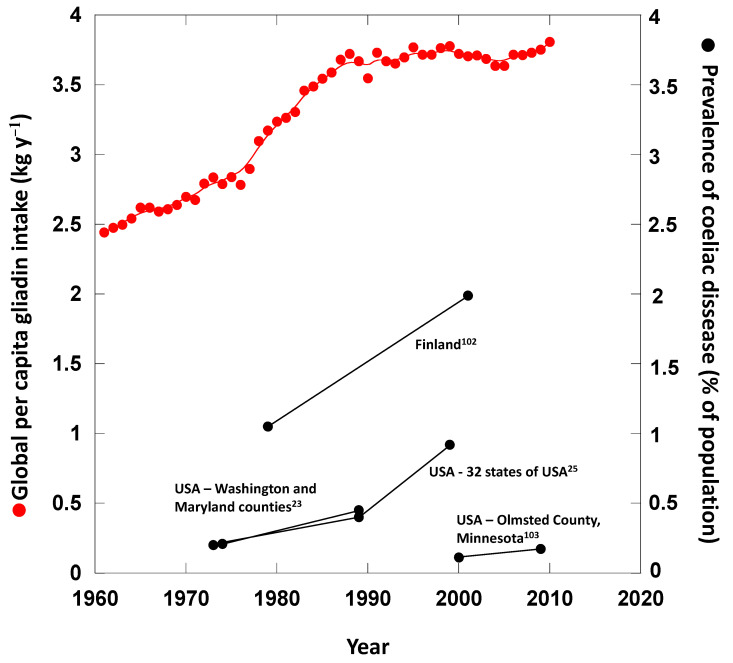
Changes in global per capita intake of gliadins, global per capita intake of gliadins per kg of N fertilisation, amount of N fertilizer applied per tonne of harvested wheat grains and the prevalence (percentage of coeliac cases in the total population) or incidence (new cases per 1000 inhabitants and year) of coeliac disease from studies with large and representative sets of population data, adjusted for age and sex, from 1961 to 2010 [23,25,102,103].

**Figure 6:**
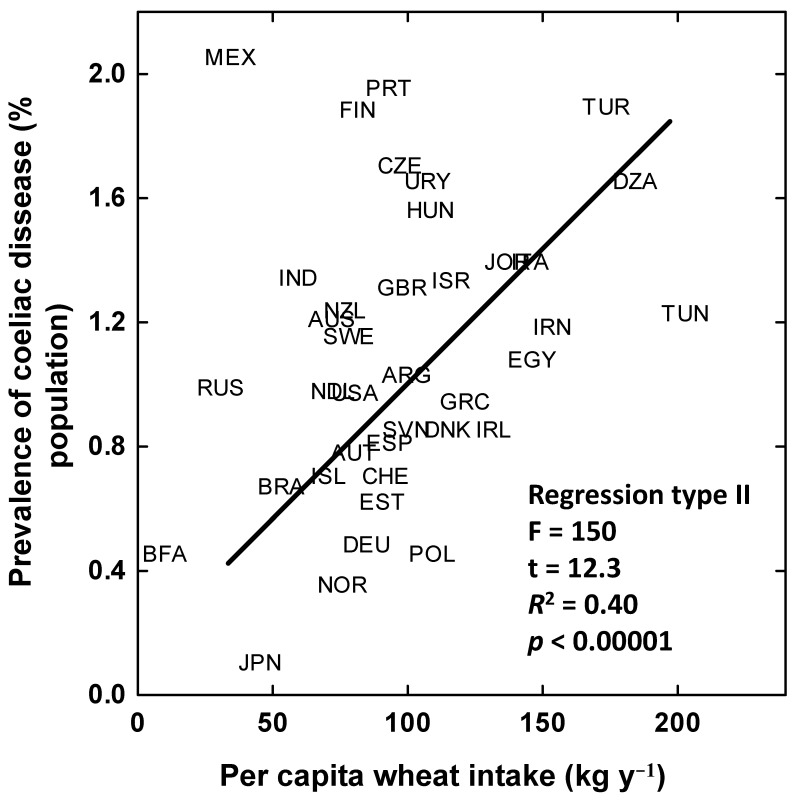
Relationship between the prevalence of coeliac disease and the per capita wheat intakes at country level. DZA = Argelia. ARG = Argentina. AUS = Australia. AUT = Austria. BFA = Burkina Faso. BRA = Brazil. CZE = Czech Republic. DNK = Denmark. EGY = Egypt. EST = Estonia. CUB = Cuba. FIN = Finland. DEU = Germany. GRC = Greece. HUN = Hungary. IND = India. ISL = Iceland. IRN = Iran. IRL = Ireland. ISR = Israel. ITA = Italy. JPN = Japan. MEX = Mexico. NLD = Netherland. NZL = New Zealand. NOR = Norway. POL = Poland. PRT = Portugal. RUS = Russia. SVN = Slovenia. ESP = Spain. SWE = Sweden. CHE = Switzerland. TUN = Tunisia. TUR = Turkey. GRB = United Kingdom. USA = United States of America. URY = Uruguay. Data from [3,15,104,105,106,107,108].

A comparison of the changes in global per capita intake of gliadins from 1961 to 2010, with data for CD prevalence (percentage of coeliac cases in the total population) or incidence (new cases per 10,000 inhabitants per year) provided by studies of large and representative sets of population data, adjusted for age and sex is shown in Figure 5. The increases in prevalence/incidence during this period coincided with the per capita increase in gliadin intake associated to the increase in the application of N fertilizer per tonne of wheat grains produced and with the per capita increase in gliadin intake (Figure 5). The higher per capita ingestion of gluten/gliadin globally in recent decades could thus account for, at least partially, the spread of coeliac pathology in the global human population. New research is though warranted to test this possibility and to figure out why instead the prevalence of CD is comparable between countries in which the intake of gluten is much higher, e.g., Italy with its high consumption of pasta, than in other EU countries where the intake is much lower [100]. Long-term evolutionary adaptation may play a role there. However, several studies have demonstrated the link between probability of coeliac disease development and gluten intake. For example, Makharia et al. [101] observed that northern Indian populations had much higher rates of CD than southern Indian populations despite having similar predisposing HLA susceptibility genes across the country. This was primarily attributed to the mainly wheat-based diet in the North and the rice-based diet in the South. We have checked the available data at country level for the period 2001–2017 and found a positive relationship between prevalence of coeliac disease and per capita wheat intake across 38 countries (Figure 6).

The modern procedures of management and processing (shortening the fermentation time, use of non-acid dough, add protein fortification and use of refined white flour) could have also increased the exposure to immunoreactive compounds [109]. The use of different genotypes of wheat may be involved too since the gluten and gliadin composition are highly determined by environmental effects but also by genetic differences among wheat varieties. The genotypes and varieties of cultivated wheat have also changed in these last 60 years. Breeding practices in wheat genotypes have been mainly addressed to achieve higher yield, rheological conditions and gluten quality and high protein concentration for better baking and also for better livestock foods [7,110,111], providing stronger glutens rich in glutenins [89,112] but with scarce effects on gliadins and coeliac disease prevalence [10].

Some studies have reported that breeding activities in the last decades have contributed to increase the prevalence of coeliac disease [113], but there are more studies reporting no contribution of modern wheat breeding practices to coeliac disease prevalence during the last decades [109,112,114]. Most studies have reported that both modern and ancient wheat genotypes have similar concentrations of the pathogenic peptides responsible of inflammatory diseases [115,116,117,118,119,120,121,122], similar quantities of immunostimulatory epitopes [109,115,119,123], similar human T cell immunological responses [124,125,126,127] and similar immunogenic peptide sequences [128]. Some studies have even concluded that old varieties produced larger amounts of peptides containing immunogenic and toxic sequences than modern ones [129] and also that old varieties trigger more inflammatory processes in gut [130]. The current literature does not thus sustain the hypothesis that the shift in wheat genotypes could be a significant potential explanation for the rise in coeliac disease at global scale. Furthermore, breeding is now aiming to produce new wheat genotypes with less gliadin epitopes but without clear success yet [30]. The research to find wheat genotypes with less capacity to produce coeliac disease, i.e., less immune-reactive wheat genotypes, is very recent [116]. Independently of wheat subspecies, our results show that the increase in N fertilisation is related with increasing levels of gluten and gliadin concentrations in wheat grain and flour in all types of wheat genotypes and varieties studied, that the average per capita intake of wheat flour and grain food derivatives has been kept more or less constant in the last 60 years, and therefore the per capita ingest of gluten and gliadins has substantially increased.

There are, moreover, other possible factors predisposing to the development of CD such as the many substances emitted by humans into the environment. For instance, some studies have reported significant relationships between the ingestion of glyphosate, an increasingly used herbicide, and the predisposition to the development of CD [39,131]. In fact, CD is a very complex pathology, whose development involves not only environmental factors (gluten) but also genetic factors. Recently, a gene Inc13 has been identified that encodes for a noncoding RNA that blocks and represses the expression of certain inflammatory genes under normal conditions. The Inc13 expression can be inhibited by stimulation and increased expression of inflammatory genes favoring CD [132]. Moreover, the inflammatory over-expression of T cells could be also favoured under the infestation of certain reovirus that would suppress peripheral regulatory T cell [133]. In any case, though, we now know that autoimmune responses are generally triggered by gliadin peptides [39], and we have shown here that these gliadin peptides increase their concentration in grains in response to the intensification of N fertilisation (Figure 2 and Figure 3).

## 5. Conclusions

The intensification of N fertilisation of wheat crops has been very high (ten-fold since 1961). Our meta-analysis of the literature has demonstrated that wheat growing under higher soil N availability produces not only higher yield but also grains and flours with higher gliadin concentrations in all type of wheat genotypes. Since gliadins are the main direct responsible triggering coeliac disease and the per capita intake of wheat products in the last decades has remained more or less constant, there has been a rise in per capita intake of gliadins at the global scale. We suggest that the rise in coeliac disease reported in several human populations around the world could be related, at least in part, to N fertilisation intensification of wheat crops. If this suggested link between N fertilisation intensification and coeliac disease expansion is demonstrated in future experimental studies, we will have an important tool to control and prevent the expansion of coeliac disease.
